# Color‐Tuning Mechanism of Electrically Stretchable Photonic Organogels

**DOI:** 10.1002/advs.202202897

**Published:** 2022-07-07

**Authors:** Jun Hyuk Shin, Ji Yoon Park, Sang Hyun Han, Yun Hyeok Lee, Jeong‐Yun Sun, Su Seok Choi

**Affiliations:** ^1^ Department of Electrical Engineering Pohang University of Science and Technology (POSTECH) 77 Cheongam‐Ro, Nam Gu Pohang Gyeongbuk 37673 Republic of Korea; ^2^ Department of Materials Science and Engineering Seoul National University Seoul 08826 Republic of Korea; ^3^ Research Institute of Advanced Materials Seoul National University Seoul 08826 Korea

**Keywords:** Bragg diffraction, dielectric elastomer actuators, electrically stretchable organogels, photonic band structures, photonic crystals, photonic organogels, wavelength tuning

## Abstract

In contrast to nano‐processed rigid photonic crystals with fixed structures, soft photonic organic hydrogel beads with dielectric nanostructures possess advanced capabilities, such as stimuli‐responsive deformation and photonic wavelength color changes. Recenlty, advanced from well‐investigated mechanochromic method, an electromechanical stress approach is used to demonstrate electrically induced mechanical color shifts in soft organic photonic hydrogel beads. To better understand the electrically stretchable color change functionality in such soft organic photonic hydrogel systems, the electromechanical wavelength‐tuning mechanism is comprehensively investigated in this study. By employing controllable electroactive dielectric elastomeric actuators, the discoloration wavelength‐tuning process of an electrically stretchable photonic organogel is carefully examined. Based on the experimental in‐situ response of electrically stretchable nano‐spherical polystyrene hydrogel beads, the color change mechanism is meticulously analyzed. Further, changes in the nanostructure of the symmetrically and electrically stretchable organogel are analytically investigated through simulations of its hexagonal close‐packed (HCP) lattice model. Detailed photonic wavelength control factors, such as the refractive index of dielectric materials, lattice diffraction, and bead distance in an organogel lattice, are theoretically studied. Herein, the switcing mechanism of electrically stretchable mechanochromic photonic organogels with photonic stopband‐tuning features are suggested for the first time.

## Graphical abstract

The color‐tuning mechanism of electrically stretchable organogels is investigated in comparison with mechanical stretching method for the first time. The hexagonal close‐packed (HCP) nanostructure changing is analyzed using Bragg–Snell conditions with in situ wavelength observations. In contrast to the lattice deformation in mechanically stretched uniaxial mechano‐chromic process, the lattice stability of HCP structured organogels in electrically stretched mechano‐chromic mechanism is reported.

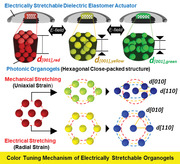

## 1 Introduction

Light propagation control using photonic crystals with periodically repeating dielectric structures has attracted considerable research interest for various photonic applications, such as color excitation,^[^ ^1^, ^2^, ^3^, ^4^, ^5^ ^]^ imaging,^[^ ^6^, ^7^, ^8^, ^9^ ^]^ lasers,^[^ ^10^, ^11^, ^12^ ^]^ and optical sensors.^[^ ^13^, ^14^, ^15^, ^16^ ^]^ Using a sophisticated nano‐patterning process to repeat periodic spatial arrangements in dielectric materials, artificial 1D,^[^ ^17^, ^18^, ^19^, ^20^, ^21^ ^]^ 2D,^[^ ^22^, ^23^, ^24^ ^]^ and 3D^[^ ^25^, ^26^, ^27^ ^]^ photonic forbidden band structures^[^ ^28^, ^29^, ^30^, ^31^, ^32^ ^]^ can be obtained. However, colored light propagation control is found in various natural self‐organized dielectric nano‐periodic structures with large areas, in contrast to artificial nanophotonic structures with limited scalability; these naturally occurring structures can also control their photonic wavelength.^[^ ^33^, ^34^, ^35^, ^36^, ^37^ ^]^ Therefore, the facile reproduction of repeating dielectric structure periodicities and effectively controlling the photonic wavelength are key research topics in the creation of artificial photonic systems that mimic those nano periodicity in nature.

Unlike nano‐scale processes for patterning dielectric structures^[^ ^38^, ^39^ ^]^ in 1D and 2D,nano‐sized dielectric beads included in 3D photonic structures that mimic natural systems such as opal can be artificially reproduced in self‐assembly processes.^[^ ^40^, ^41^, ^42^, ^43^, ^44^, ^45^ ^]^ Moreover, when nano‐spherical dielectric periodicities are introduced in soft polymeric materials, elastic structure deformation can be realized using various triggering methods.

Consequently, stimulus‐responsive mechanochromic color change is obtainable via the chemical,^[^ ^46^, ^47^, ^48^, ^49^ ^]^ light,^[^ ^50^ ^]^ magnetic,^[^ ^51^, ^52^ ^]^ electrical,^[^ ^24^, ^53^ ^]^ and thermal control^[^ ^54^, ^55^, ^56^ ^]^ of an organogel lattice. Therefore, configuring the elastic mechanochromic lattice of an organogel can overcome the major technical challenges obstructing an easy reproduction process and can dynamically control the emission wavelength of artificial photonic crystals.^[^ ^25^, ^57^, ^58^, ^59^ ^]^ Intensive research in this field has promoted the understanding of the color switching mechanism in mechanically stretchable organogels.^[^ ^60^, ^61^ ^]^ Particularly, the color switching mechanism of a uniaxially strained photonic gel lattice in a mechanochromic system was well investigated and reported.^[^ ^62^, ^63^, ^64^, ^65^, ^66^, ^67^, ^68^, ^69^ ^]^

An electric‐based color control method is ideally desired for maximizing the practical applicability of such hydrogels. The proposal of such electric‐oriented strategies in the past has been limited, albeit for a recent study that demonstrated color‐changing photonic organogels using an electroactive dielectric elastomer actuator (DEA).^[^ ^2^, ^43^, ^70^, ^71^, ^72^ ^]^ Nevertheless, the wavelength‐tuning mechanism of such electrically stretchable organogels is not well understood yet. Although recent reports have comprehensively illustrated the uniaxial mechanical stretching‐induced color change mechanism of similar hydrogels,^[^ ^73^, ^74^, ^75^, ^76^, ^77^, ^78^, ^79^, ^80^ ^]^ a deeper understanding of electrically stretchable chromonic organogel systems, in comparison with mechanical stretching mechanochromic systems, is required. However, elucidating the electrical mechanochromic processes involved in electrically stretchable and variable nanostructures, presents notable technical challenges. For example, the direct in‐situ observation of dynamic nanolattice changes under electrically induced strain is severely limited.^[^ ^58^, ^59^, ^60^, ^61^, ^62^, ^63^, ^64^, ^65^, ^66^, ^67^, ^68^, ^69^ ^]^ Moreover, the strain directions applied to the nanostructure of complex electrically stretchable nano organogels are typically multiple and complex, in contrast to the uniaxial strain applied during the direct mechanical stretching of mechanochromic systems. Thus, it is crucial to develop an alternative approach that facilitates the direct in‐situ observation of structural changes in the organogel nanolattice under the application of external electric fields and complex strains. An important aspect during the experimental analysis of electrically stretchable nanophotonic organogels is to accurately reflect in situ dynamic wavelength change phenomenon. Therefore, establishing a theoretical model that correlates the nanostructural changes with the shifting photonic output, while illustrating in‐situ the wavelength change of the nano lattice under electrical stretching stress, is highly desired.

In this study, we investigated the theoretical mechanism underlying the nanophotonic lattice changes in an electrically stretchable photonic organogel, while illustrating the evolution of the photonic wavelength shift process. In consideration of the working behavior of DEAs, the electrically controlled mechanochromic photonic stopband shifts were comparatively investigated with simple mechanical‐stretching‐based color change methods. The mechanism underlying the electrically stretchable mechanochromic wavelength change process in a nanoelastic photonic organogel was thoroughly discussed and elucidated in accordance with the Bragg‐Snell model. More importantly, the triggering mechanism responsible for the nanophotonic lattice structure shifts of a hexagonal close‐packed (HCP) stretchable organogel was proposed for the first time.

## 2 Results and Discussion

**Figure 1:**
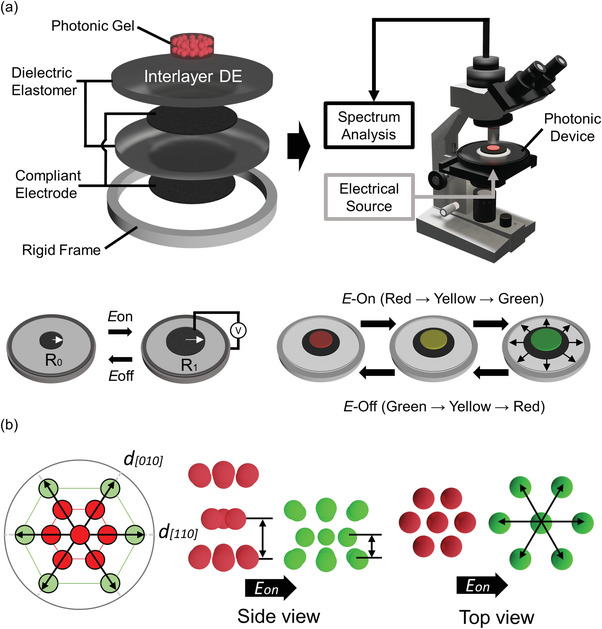
Schematic of electromechanical stretching‐induced color change of photonic organogels. a) Observation system of electrically stretching mechanochromic gels with in‐house optical microscopic setup and electromechanical stretcher. The DEA was sandwiched between the circular top and bottom compliant electrodes. The active area of symmetrical expansion was calculated according to the diameter difference between *R*_0_ (initial diameter at the E‐Off state) and *R*_1_ (expanded diameter at the E‐On state). b) Color‐changing mechanism of electromechanically stretchable mechanochromic photonic gel.

**Figure** **1** illustrates a summary of the color switching mechanism of an electroactive photonic organogel under electromechanical stretching. First, to better understand the electrically induced mechanochromic color‐tuning mechanism, an electroactive photonic organogel was prepared, and its electrooptical behavior was carefully observed. The electromechanical stretching‐induced color change behavior of our mechanochromic organogel was compared with other reported mechanical stretching‐based color change mechanisms using an in‐house polarized microscopic setup, that comprised a mechanical stretching system and a separated optical microscope for electrical signal‐based stretching (Figure 1a). Each optical setup was connected to a computer with spectroscopy and an optical complementary metal‐oxide semiconductor (CMOS) camera. The in‐house optical microscope setup is described in Figure S1, Supporting Information.

The electromechanical stretching‐induced color change of the organogel should be ideally powered by the electro‐active stretching of a DEA. Therefore, an electroactive DEA was fabricated with a pre‐stretched dielectric elastomer (DE; VHB 4905, 3M)^[^ ^81^ ^]^ to induce electromechanical deformations on the photonic organogel. To electrically trigger the soft actuator, the pre‐stretched DEA was fixed to a rigid frame and sandwiched between a top and bottom circular compliant electrode (Carbon grease, MG Chemicals). Additionally, a circular photonic organogel was attached to the active electrode area using an adhesive DE interlayer. Self‐assembled polystyrene (PS) beads containing nanophotonic organogel were prepared according to a previous report.^[^ ^43^ ^]^ When an electric field was applied, electromechanical stretching deformation was induced with symmetrical radial expanding of DEA actuations (Figure 1a). This symmetrical stretching strain, with subsequent photonic reflection, resulted in a color change starting from red to green, and then blue. Note that the electromechanical stretching of the photonic organogels was directly affected by the symmetrical expanding actuations of the DEA with the contraction of thickness. In recent studies where similar mechanochromic systems were investigated,^[^ ^70^, ^73^, ^76^, ^78^, ^79^ ^]^ the organogels exhibited hexagonal lattice structures. However, in contrast to the uniaxial deformation of the HCP lattice observed in these studies, here, the HCP lattice structure of the electrically stretchable nano organogels with circular compliant facing electrodes was symmetrically expanded along both the horizontal and vertical directions during the color change process. It was thus inferred that the electromechanical stretching treatment caused the hexagonal lattice of the organogels studied here to symmetrically and radially expand and contract along both the vertical and horizontal directions (Figure 1b).

**Figure 2:**
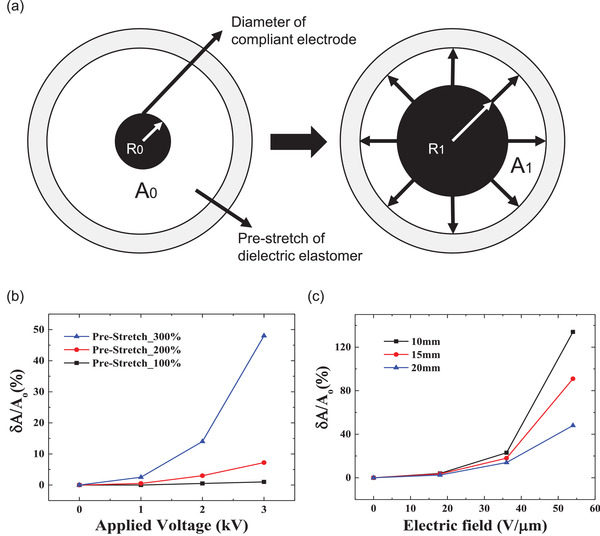
Effects of DEA film pre‐stretch ratio and compliant electrodes size. a) Difference of DEA actuation with regard to the pre‐stretch ratio and the area of the compliant electrodes under an externally applied electric field. b) DEA areal strain curves under pre‐stretching conditions of 100%, 200%, and 300%. c) Dependency of DEA areal strain on the compliant electrodes diameter (*R*_0_ = 10, 15, 20 mm) for the 300% pre‐stretching condition.

To better determine the ideal initial electrical stretching power, the optimum DE pre‐stretching amount was investigated (**Figure** **2**). To optimize the stretching power of the DEA, its electrical actuation was carefully monitored as a dual function of the applied electric field and the biaxially pre‐stretched amount, by observing the shape changes of the compliant electrodes. It should be noted that the elastic actuation force of the DEA can increase due to pre‐stretching; therefore, the stretching displacement power of the DEA should be investigated and pre‐determined before placement on the photonic organogel.

When the DEA was actuated with electric voltage, the active area between the circular shaped electrodes expanded and showed stretching behavior (Figure 2a). Therefore, the DEA‐induced displacement can be defined by *δA/A*_0_, where *A*_0_ is the initial active area of the DEA at the E‐Off State, *A*_1_ is the expanded actuating area of the DEA at the E‐On state, and *δA* is the shape change of the active area (*δA = A*_0_ *− A*_1_). The dynamic shape change of the active electrode was measured in real time using a laser displacement sensor (LJ‐V7200, Keyence). The pre‐stretching amount of the DEA was varied at 100%, 200%, and 300%, and the active electrode shape change was radially expanded along the lateral direction as a function of the applied electric voltage. The DEA active area expansion was maximized under the highest pre‐stretching conditions of 300% (*δA/A*_0_ = 48%), compared to 100% (*δA/A*_0_ = 2.5%), and 200% (*δA/A*_0_ = 14%) (Figure 2b). Thus, it was inferred that the electromechanical stretching in terms of (*δA/A*_0_), which denotes the actuating lateral expansion of the electrode area (*δA*) compared to the initial electrode area (*A*_0_) at the E‐Off state, showed an upward tendency as the biaxial pre‐stretch amount was increased to 300%. The actuating stretching of the DEA positively increased as a function of the applied electric voltage. Additionally, the DEA film was more prone to tearing by pre‐stretching above 300%.

In addition, it was observed that the actuating stretching power was stronger when the initial compliant area in terms of *A*_0_ was smaller. Further, it was discovered that a relatively smaller *R*_0_ (initial active electrode area at the E‐Off state under a fixed rigid frame), combined with a larger *R*_1_ (circular expanded radius at the E‐On state), led to a larger *δA/A*_0_ actuating expansion power. The actuating stretching change in terms of *δA/A*_0_ was maximum when the initial circular radius (*R_0_*) was minimum (=10 mm) and the applied electric field intensity was at maximum. It was theoretically assumed that the relatively large viscoelastic space of the DEA (between the active region and the fixed frame) enabled its further lateral areal expansion.

Consequently, a biaxially 300% pre‐stretched DEA film, sandwiched between top and bottom circular compliant electrodes with a radius of 10 mm, was prepared as the optimum electrically stretching actuator (Figure 2c).

**Figure 3:**
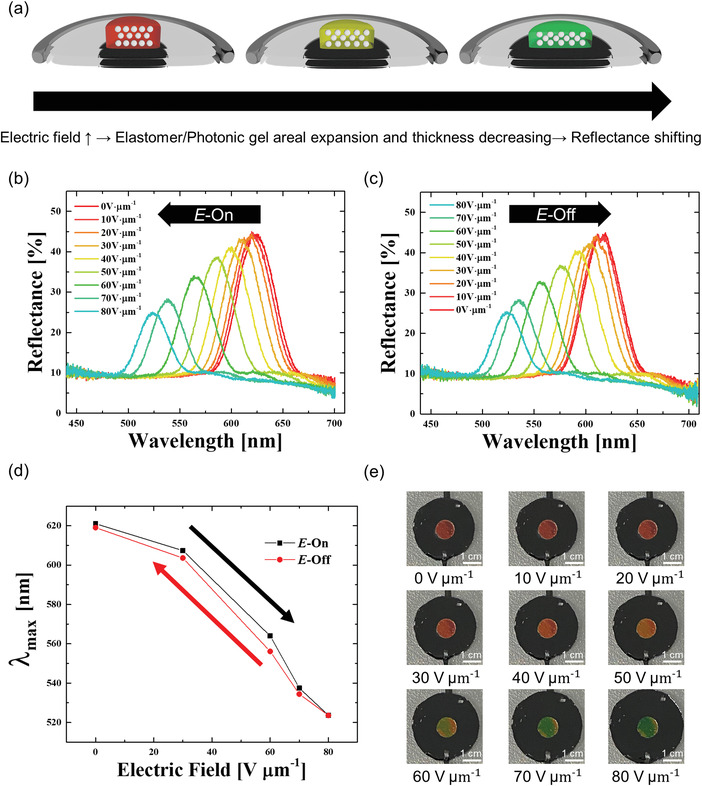
Electromechanical stretching behavior of the photonic organogel. a) Electroactive mechanochromic color change. b) Stretching wavelength shift of the electric lateral expansion mode at the E‐On state. c) Recovering wavelength shift of the electric lateral compression mode at the E‐Off state. d) Monitoring plot of the electroactive mechanochromic wavelength change in terms of photonic reflection maximum, *λ*_max_, and as a function of electric field sweep. e) Macroscopic images of the electroactive photonic organogel at different electric field intensities: 0, 10, 20, 30, 40, 50, 60, 70, and 80 V µm^−1^.

In contrast to simple mechanical stretching conditions, it is technically more challengeable to directly observe the in‐situ changes of an organogel's structure under electromechanical stretching. Therefore, an indirect approach to understand this electrically stretchable organogels system but can reflect experimental phenomenon of this electrically stretchable color change. Therefore, to directly observe the experimental in situ response of electrically stretchable photonic organogels, the resulting output of an electrically induced wavelength shift process was carefully examined (**Figure** **3**). Upon the application of an electric field, the areal lateral expansion of the DEA‐induced electromechanical color shifts to the photonic organogels, similar to the pure mechanochromic behavior. The lateral expansion of the DEA upon the application of an electric field resulted in the lateral stretching of the photonic organogel, causing continuous color change from red to green. The color of the organogel returned from green to red in the lateral compression mode of the DEA with the decreasing electric field (Figure 3a and Video S3, Supporting Information).

The reflectance spectra were blue shifted as a result of the electric field, while retaining the optical quality. Specifically, it was discovered that the *λ*_max_ (wavelength at maximum reflectance) of the reflection photonic band shifted from 621.02 to 619.84, 619.64, 607.37, 600.41, 587.66, 563.98, 537.47, and 523.62 nm when the electric field ranged from 0 to 80 V µm^−1^ in increments of 10 V µm^−1^, respectively (Figure 3b). The tuned photonic band spectrum of the mechanochromic organogel was recovered to its original wavelength position as the applied electric field was decreased (Video S4, Supporting Information). The reflecting *λ*_max_ was fully recovered to 523.62, 534.42, 556.11, 574.84, 589.85, 603.60, 609.55, 611.33, and 619.05 nm as the electric field decreased from 80 to 0 V µm^−1^ in decrements of 10 V µm^−1^, respectively (Figure 3c). The reversible electrooptical behavior of the electrical mechanochromic organogel was highlighted by plotting the constant reflection *λ*_max_ of the photonic band in either the lateral expanding or lateral compression mode (Figure 3d). The same macroscopic reflection color was observed at the same applied electric field conditions in either mode (Figure 3e). It should be noted that minimal photonic tuning wavelength hysteresis was observed during the electrical stretching process, as shown in Figure 3d. This switching hysteresis of the electrically stretchable wavelength contradicted the vivid hysteresis of the conventional mechanical stretching of organogels (Figure S3c , Supporting Information). This was assumed to have been caused by the stepwise electric field change, which allowed the full recovery of the structure, in contrast to the mechanical deformation process of full stretching and relaxation with radical elastic inertia. Considering that the wavelength switching hysteresis phenomenon usually stems from structural disparities in the organogel nanolattice, the electrical stretching strategy could effectively maintain the lattice structure during the stretching and relaxation stages (as opposed to the mechanical stretching process). More importantly, electrooptical reversibility was also confirmed from simultaneous observations of the dynamic mechanochromic behavior (Video S3, Supporting Information).

**Figure 4:**
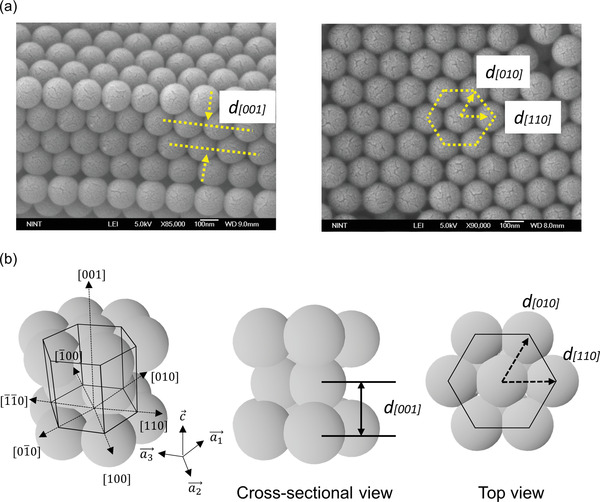
Lattice model of the closely packed PS beads. a) SEM image of the closely packed PS beads in cross‐sectional and top views. b) Illustration of the HCP lattice structure model with *d*_001_, *d*_010_, and *d*_110_ distances.

From field emission‐scanning electron microscope (FE‐SEM) observations, the self‐assembled PS bead nanostructure in the photonic organogel was found to form an HCP structure (**Figure** **4**a). Considering the lateral expansion with the thinning of the photonic organogel through the application of a vertical electric field, it can be assumed that the nano‐packed structure of the PS beads would expand along the [010] and [110] planes, whereas the thickness along the [001] plane decreases during the mechanochromic tuning process (Figure 4b).

Therefore, the closely packed PS nano‐beads in the photonic organogel successfully formed an HCP structure during the self‐assembly process in this study. Because the two different refractive indexes of the elastic organic gel (*n*_1_ = 1.479) and PS (*n*_2_ = 1.59) were periodically combined in the photonic organogel, the observed color change due to the photonic wavelength band shift can be explained using the Bragg–Snell model. In the top‐view observation of the sample, the Bragg–Snell model should be analyzed along the [001] direction. During the symmetrical and lateral expansion of the organogel by the electrical stretching of the DEA, it was assumed that the distances between the adjacent PS beads in the [010] and [110] planes (respectively denoted as *d*_010_ and *d*_110_) were increased, whereas *d*_001_ (=the distance of the adjacent PS beads in the [001] direction) corresponded to the thinning of the lattice and the blueshift of the Bragg–Snell reflection (Figure 4b).

The light reflectance from the photonic organogel was determined by the arrangement of the PS beads in the HCP lattice. Therefore, the electromechanical stretching‐induced wavelength changes of the photonic organogels discussed in Figure 3 were inferred to result from the in‐situ changes in the HCP nanolattice structure (Figure 4). Considering our in‐house microscope setup, where the vertical incident light was parallel to the normal direction of the photonic organogel, the lattice distance variation along the *d_001_* direction was calculated using the modified Bragg–Snell diffraction conditions^[^ ^82^ ^]^

(1) $$d_{001}=\frac{\lambda_{\max}}{\sqrt{\sum_{i}n_{i}^{2}\cdot V_{i}^{\prime }}}\times \sqrt{\frac{3}{8}}$$

(2) $$\sum_{i}n_{i}^{2}\cdot V_{i\;}^{\prime }=\;n_{1}^{2}\cdot V_{1\;}^{\prime }+\;n_{2}^{2}\cdot V_{2\;}^{\prime }$$

where *λ*_max_ denotes the wavelength of the reflectance band maxima, and *d*_001_ denotes the assumed lattice distance during the wavelength tuning of *λ*_max_ in the organogel. The refractive indexes of the organogel components were considered as *n*_1_ = 1.479 for the elastic organic gel (volume fraction *V*_1_ = 0.26), and *n*_2_ = 1.59 for the rigid nano‐scale PS beads (volume fraction *V*_2_ = 0.74).

**Figure 5:**
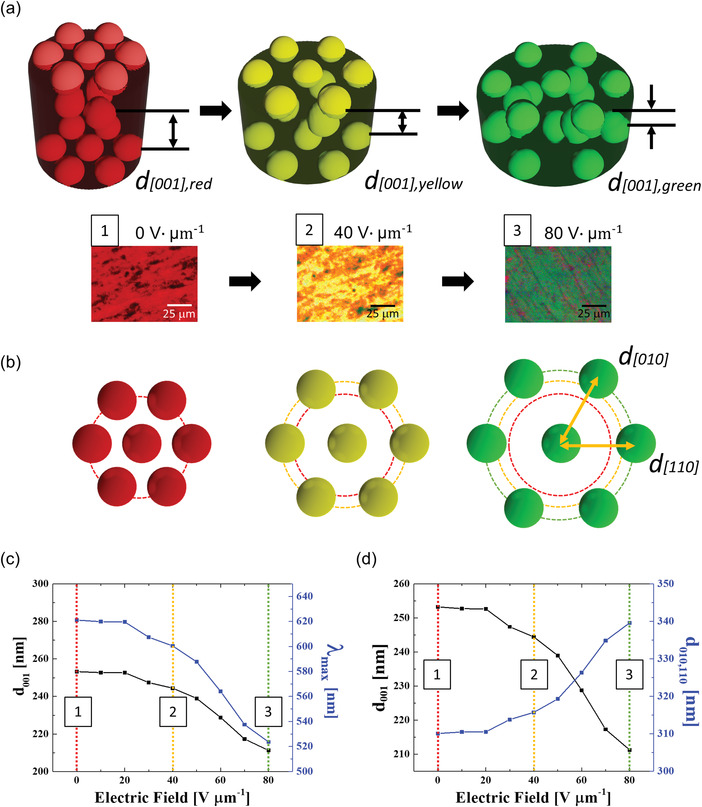
Behavior of electrically stretchable photonic organogel lattice under an electric field. a) Changes in the HCP lattice under the application of an electric field and corresponding microscopic images (200 × magnification) of the photonic organogel surface under different electric field intensities (0, 40, 80 V µm^−1^). b) Changes in the PS beads structure in the lateral [110] and [010] directions. c) Decreasing trends of *d*_001_ and *λ*_max_ under the application of an electric field. d) Symmetrical trade‐off of horizontal lattice distance change as a function of electric field.

Since the closely packed PS beads were isotropically swollen by the organogel,^[^ ^83^, ^84^, ^85^ ^]^ *V*_1_ and *V*_2_ could be changed to *V*_1_’ and *V*_2_’. The lattice volume between the *d*_001_ spaces became larger due to ultraviolet irradiation increasing the stiffness of the organogel. The volume fraction of the PS beads in the photonic organogel could be calculated by the HCP unit volume of the photonic organogel divided by the total volume of the PS beads, as explained below

(3) $$V_{2\;}^{\prime }=\;V_{2}\;\times \;{\left(\frac{2r}{d_{010}}\right)}^{3}$$

(4) $$d_{001}=\frac{\sqrt{6}}{3}\;d_{010}\;\left(or\;d_{110}\right)$$

(5) $$V_{1}^{\prime }=1-\;V_{2\;}^{\prime }$$

(6) $$n_{1}^{2}\cdot V_{1\;}^{\prime }\cdot d_{001}^{3}-\;\frac{3}{8}\cdot \lambda_{\max}^{2}\cdot d_{001}+\;{\left(\frac{2r\sqrt{6}}{3}\right)}^{3}\cdot n_{2}^{2}\cdot V_{2\;}^{\prime }=0$$

where *r* denotes the radius of the PS beads (*r* = 100 nm). By incorporating the calculations from Equations (2), (3), (4), and (5) into Equation (1), it was revealed that the *d*_001_ lattice distance was calculated to be the solution of Equation (6), which is the cubic equation for *d*_001_. According to the calculations, the *d*_001_ lattice distance was decreased by approximately ∆*d*_001_ = 42 nm (from 253.25 to 211.25 nm), resulting from the applied electric field, which was in agreement with both the band shift in terms of *λ*_max_ (**Figure** **5**d), and the microscopic reflection changes (Figure 5c). The self‐assembled photonic lattice structure of the close‐packed PS beads was observed via FE‐SEM image (Figure 4a), which confirmed the HCP features of the nanophotonic lattice structure. Moreover, the solution infiltration process was conducive to the preservation of the HCP structure in the swollen organogels.

Accordingly, the initial adjacent PS lattice distances were calculated by the edges of the tetrahedron shape, whose height was *d*_001_ in the HCP structure.

(7) $$V_{\mathrm{initial}}=\;V_{\mathrm{stretched}}$$

(8) $$\begin{array}{ccc} & & \frac{\sqrt{3}}{4}\cdot d_{010,\mathrm{initial}}^{2}\times \;6\;\times \;2d_{001,\mathrm{initial}}\\ & & \;=\frac{\sqrt{3}}{4}\cdot d_{010,\mathrm{stretched}}^{2}\times \;6\;\times \;2d_{001,\mathrm{stretched}} \end{array}$$

(9) $$d_{010,\mathrm{stretched}}=\;\sqrt{\frac{d_{010,\mathrm{initial}}^{2}\times d_{001,\mathrm{initial}}}{d_{001,\mathrm{stretched}}}}$$

Since the DEA expanded in an anisotropic manner, the PS beads along the six lateral plane directions in the HCP structure were symmetrically expanded in a radially stretching manner. Therefore, only two out of the six HCP lateral planes, namely [010] and [110], were chosen for further investigations (Figure 4b). When the DEA was radially expanded along the lateral HCP plane, the unit volume of the HCP photonic organogel remained the same. Because the volume in the initial state was the same as the volume in the electrically stretched state (Equations (7) and (8)), *d*_001_ decreased, whereas *d*_010_ and *d*_110_ increased. The increased value of *d*_010_ and *d*_110_ can be calculated by the unit volume of the HCP photonic organogel divided by *d*_001_, and the edge of the regular hexagon was revealed to be equal to the *d*_010_ and *d*_110_ values (Equation (9)).

As a result, the initial *d*_010_ and *d*_110_ lattice distances were calculated at 310.06 nm, and increased approximately by ∆*d*_010_ = 29.54 nm (from 310.06 to 339.6 nm) (**Table** **1**).

**Table 1:** Reflective maxima (*λ*_max_) and lattice distances (*d*_001_, *d*_010_, and *d*_110_) of electrically stretchable mechanochromic organogel under different electric fields (0, 20, 40, 60, and 80 V µm^−1^)

	Electric field [V µm^−1^]				
	0	20	40	60	80
*λ*_max_ [nm]	621.02	619.83	600.41	563.98	523.61
*d*_001_ [nm]	253.25	252.66	244.43	228.77	211.25
*d*_010_ or *d*_110_ [nm]	310.06	310.52	315.71	326.33	339.6

**Figure 6:**
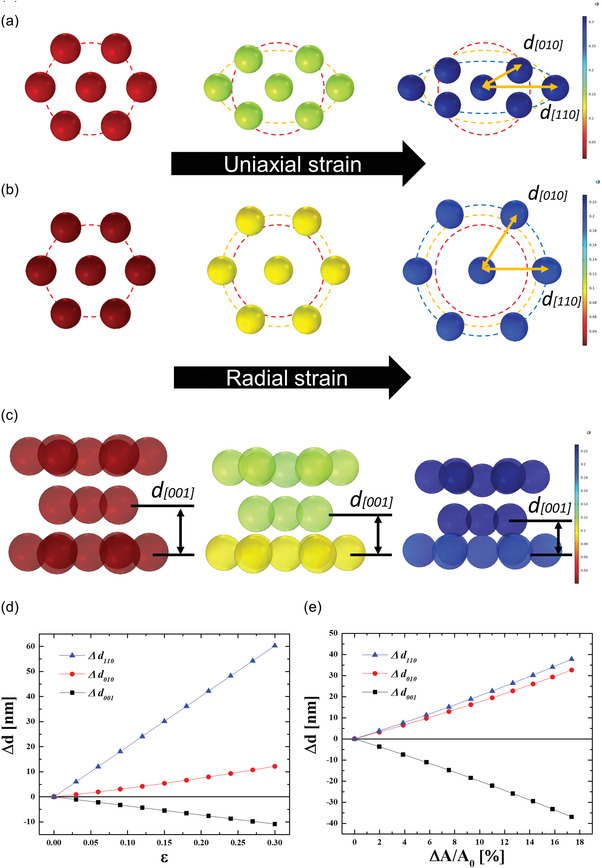
Comparative FEA investigation of HCP lattice changes under mechanical and electromechanical stretching. a) HCP lateral plane of PS beads under uniaxial strain. b) HCP lateral plane of PS beads under radial strain. c) HCP horizontal plane of PS beads along the [001] direction. Distance difference along the [010], [110], and [001] directions under d) uniaxial strain and e) radial strain.

To better determine the movement of the PS beads in the HCP lattice under uniaxial and radial stretching, we employed a finite element analysis (FEA) method. Τhe device shown in Figure S4 induced radial strain deformations on the photonic organogel by applying an electric field. Notably, when uniaxial strain was induced on the photonic organogel via mechanical stretching, it assumed an oval shape. When we focused on the HCP model unit of the PS beads in the photonic organogel, the orientations of the adjacent PS beads along the [010] and [110] directions were asymmetric (**Figure** **6**a); therefore, when uniaxial strain was induced, the strain distribution was not uniform, with the strain value showing a decreasing trend from the edge to the center of the elastomer. This asymmetrical mechanochromic characteristic of strain distortion in the photonic nanocrystal was in good agreement with a recent report.^[^ ^73^ ^]^ Uniaxial strain via direct mechanical stretching is expected to cause distortions in the symmetrical HCP lattice parallel to the strain direction; thus, the uniaxial distortion of the HCP nano lattice should lead to asymmetrical changes of the stretchable photonic gels. However, the PS beads exhibited symmetric structures along the [010] and [110] directions (Figure 6b), owing to the uniform strain distribution in the radially stretched active area of the elastomer under the influence of a circular‐expanding electro actuating system. Since the initial electrical actuation with circular compliant electrodes caused radial and symmetrical stretching to the photonic organogels, their nanolattice structure was not asymmetrically distorted. Despite the continuous expansion and compression of the lattice, the final lattice structure remained undistorted. Further, as seen in the cross‐section view of the HCP lattice unit of the PS beads, the radial strain caused the distance between the [001] spaces to decrease (Figure 6c).

The detailed quantitative FEA of the distances between the [010], [110], and [001] spaces in the HCP is presented in Figure 6d,e. When uniaxial strain was applied to the photonic organogel, the *∆d*_110_ (which denotes the *d*_110_ distance difference in the pre‐ and post‐strain states) increased along with the uniaxial strain amount. However, the *∆d*_010_ and *∆d*_110_ values were quite larger (Figure 6d) than that of the radially stretched photonic organogel device (Figure 6e), since the [110] plane was parallel to the direction of uniaxial strain in this model. Since the [010] direction was not parallel to the direction of uniaxial strain, the *∆d*_110_ was larger than *∆d*_010_. Further, the horizontal *∆d_001_* gap decreased because the volume of the HCP lattice unit remained constant (Equation (7)).

However, strain directions in radial strain were parallel to all the directions in HCP lateral directions, gap between *∆d*_010_ and *∆d*_110_ were smaller than that of uniaxial stretched device (Figure 6e). Condition for the decreasing *∆d*_001_ of the radial strain was same for the situation of uniaxial strain.

The uniaxial and radial stretching mechanism could be an important index for the optical quality of the dynamic tunable photonic device. In Videos S1 and S3, Supporting Information, which respectively demonstrate the uniaxial strain and electroactive strain (radial strain) process, a more uniform optical quality was exhibited in the latter case. As a result, the opal structure of the PS beads in the photonic organogel should be uniformly stretched in all HCP lateral directions when a high sensing resolution is required.

## 3 Conclusions

The photonic reflection color‐tuning mechanism of electrically stretchable mechanochromic organogels was investigated in this study. The effective influence of electrical stretching was carefully investigated to determine the optimal DEA actuating parameters to trigger the electrical color‐tuning features of the organogels. Specifically, a 300% biaxially pre‐stretched DEA equipped with compliant active electrodes with a 10‐mm radius was used to induce lateral electromechanical stretching on the organogels, resulting in continuous color change from red to green. By observing the self‐assembled nano‐bead lattice structure of the organogel and through detailed investigations of the wavelength shift, it was discovered that the photonic wavelength tuning could be continuously and accurately controlled. This continuous wavelength tuning was described using the Bragg–Snell model while considering the subtle *d*_001_, *d*_010_, and *d*_110_ changes in the organogel nanolattice during the electrical stretching treatment of the DEA. Furthermore, the reflectance wavelength control was fully reversible and repeatable. More importantly, the wavelength shift mechanism of electrically stretchable photonic organogels was experimentally illustrated in‐situ for the first time. We hope for our research to contribute to the elucidation of the symmetrical mechanochromic characteristics of electrically stretchable photonic crystals and promote their application potential in practical devices.

## 4 Experimental Section

### Synthesis of Stretchable Photonic Organogel

The self‐assembled structure consisting of nano‐spherical beads and a photonic organogel was synthesized following a previous report.^[^ ^43^ ^]^ PS beads with a diameter of 200 nm (Warrington, PA) and 3 wt% of aqueous solution were infiltrated to a 200‐µm gap. Then, the filtered precursor solution was slide‐casted at 1 mm min^−1^. Thermal air blowing was employed on the sample to obtain a self‐assembled rigid nano‐bead lattice during water‐drying by evaporation.

To obtain the elastic host of the photonic gel, a monomer mixture comprising 4.68 m acrylamide (Sigma Aldrich, A8887) and 4.65 m 2‐hydroxyethyl methacrylate (Sigma, 128 635) was added to ethylene glycol (Sigma Aldrich, 324 558), followed by adding 0.57 wt% of ethylene glycol di‐methacrylate (Sigma, 335 681) and 0.17 wt% of lithium phenyl‐2,4,6‐trimethyl benzoyl phosphonate (with respect to acrylamide). The final host precursor mixture was introduced into the self‐assembled photonic crystal nano‐bead lattice and cured in an ultraviolet chamber (Hoefer, UVC 500) for 30 min, to induce stiffening. The final obtained elastic photonic organogel film exhibited red photonic reflection.

### Fabrication of Electroactive Dielectric Elastomer Actuator

A DE (VHB 4905, 3M) was pre‐stretched with an in‐house biaxial stretching jig up to 300%. To maintain the pre‐stretched conditions, a circular donut‐shaped plastic rigid frame was placed on the biaxially pre‐stretched DE film, and the out‐of‐frame boundary was removed. To apply the electric field for electrical actuation of the pre‐stretched dielectric elastomer, 10‐mm‐diameter compliant circular electrodes (carbon grease, MG chemicals) were coated on the top and bottom surfaces of the DEA. The 300% biaxial stretching of the DEA and the 10‐mm diameter of the circular compliant electrodes were determined from an actuation study (Figure S1, Supporting Information). A laser displacement sensor (LJ‐V7200, Keyence) was also used to measure the actuating shape difference of the DEA.

### Integrated Measurement Setup

An amplified electrical signal was generated using a high‐voltage amplifier (609B‐3, Trek) and an arbitrary function generator (AFC3000C, Tektronix) while monitoring the applied signal using an oscilloscope (TBS1000C, Tektronix). By connecting the final electric signal to the top and bottom compliant electrodes, the electrooptical behavior of the photonic organogel was investigated. The magnified optical status of the sample and the spectral wavelength changes were simultaneously measured using the designed in‐house microscope (BX‐51, Olympus) equipped with an integrated spectrometer (Flame‐T, Ocean Optics) and a CMOS camera (HAWK‐SCM63, Zootos), as demonstrated in Figure S1, Supporting Information.

To realize the repeated mechanical stretching of the organogels, a controlled motorized mechanical stretching system was designed in‐house. The motorized directional stretching system was designed to be integrated with the microscope setup presented in Figure S1, Supporting Information. Real‐time observations of the spectrum and optical images were possible during the stretching and relaxing of the photonic organogels.

### Analytical Simulation of Stretchable Organogel Lattice Model

The simulation of the photonic organogel's modeling unit and the deformation pattern of the photonic crystal's arrangement were computed using the COMSOL Multiphysics 5.6 software package. The Young's moduli of the soft substrate, organogel, and PS beads in the FEA were 220 kPa, 22 kPa, and 7.9 GPa respectively. In‐plane strain was applied to the edge of the soft substrate. In the simulation of the organogels’ mechanochromic behavior, uniaxial strain was induced along the *x*‐axis; however, radial strain was induced for the analysis of the electroactive mechanochromic behavior. Subsequently, the distance of the adjacent PS beads in the horizontal and vertical plane directions was measured.

## Conflict of Interest

The authors declare no conflict of interest.

## Supporting information

Supporting Information

Supplemental Video 1

Supplemental Video 2

Supplemental Video 3

Supplemental Video 4

Supplemental Video 5

Supplemental Video 6

## Data Availability

The data that support the findings of this study are available in the supplementary material of this article.
