# Supplementary material for: Color‐Tuning Mechanism of Electrically Stretchable Photonic Organogels
Source: Adv Sci (Weinh). 2022 Jul 7;9(25):2202897. doi: 10.1002/advs.202202897 (PMC9443443; doi:10.1002/advs.202202897)
Supplement: Supplementary file 1 — Supporting Information [file ADVS-9-2202897-s003.pdf]

ADVANCED SCIENCE
Open Access

Supporting Information

for *Adv. Sci.*, DOI 10.1002/advs.202202897

Color-Tuning Mechanism of Electrically Stretchable Photonic Organogels

*Jun Hyuk Shin, Ji Yoon Park, Sang Hyun Han, Yun Hyeok Lee, Jeong-Yun Sun and Su Seok Choi**

Supporting Information

Color-Tuning Mechanism of Electrically Stretchable Photonic Gel

*Jun Hyuk Shin, Ji Yoon Park, Sang Hyun Han, Yun Hyeok Lee, Jeong-Yun Sun, and Su Seok Choi**

Electro-optical Measurement Setup for Mechano-chromic Oragnogels

Figure S1 demonstrates in-house designed electro-optical measurement system which allows concurrent investigations of optical image, spectral shift and electrical conditions in same time from the modified microscope set up (BX51, Olympus). Mechano-chromic optical response of the photonic organogel was studied using an integrated spectrometer (Flame-T, Ocean Optics) and real time collected optical image from CMOS camera (HAWK-SCM63, Zootos) on microscope under various electrical conditions. An automated mechanical stretching system was designed in-house using controlled motorized stretching jig system which can be integrated on the microscope set up. (Figure S1(a))

Also, for controlled electrical signal, an integrated function generator (AFC3000C, Tektronix) connected with a high-voltage amplifier (609B-3, Trek) and an oscilloscope (TBS1000C, Tektronix) was used. The reflectance could be measured in real time with varying the electric field from high-voltage amplifier and function generator. (Figure S1(b))

(a)

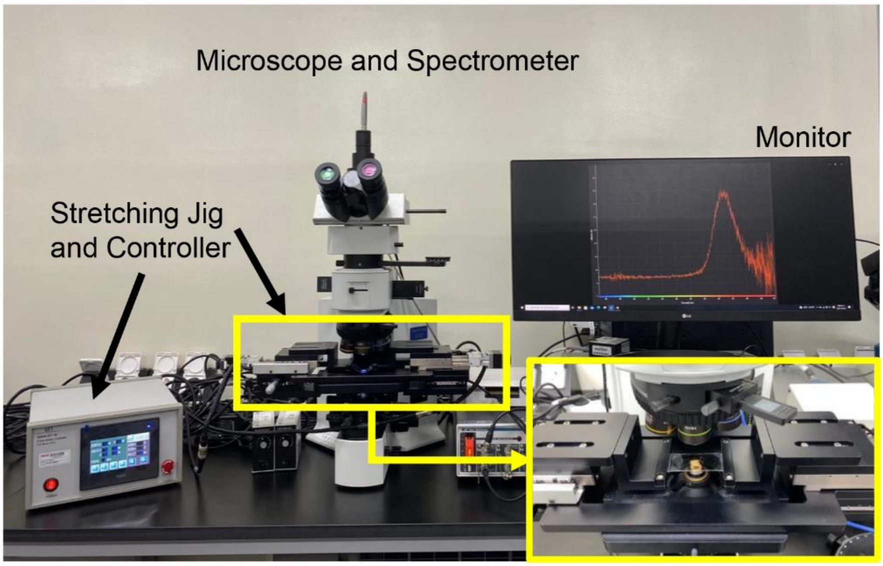

(b)

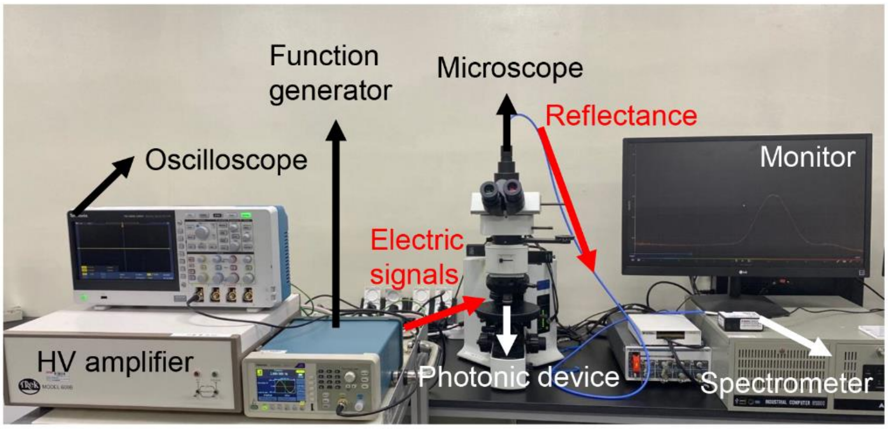

Figure S1. System of simultaneous reflectance spectrum analysis with (a) mechanically stretching system and (b) generating electric field (function generator and voltage amplifier) and microscope with spectrometer.

Repeatability of the electroactive photonic device

In further of reversible reflection change with same electric field conditions under the microscope observation (Supporting video S4), it was believed that electrical wavelength switching control of the photonic organogel can be reliable and repeatable for practical device applications. The evolutional wavelength tuning process of electro-active photonic organogel was monitored as a function of time. Upon applying electric field of 80 V μm^{-1} the reflection photonic band spectrum in normalized was blue shifted with fine time resolution, **Figure S2(a)**. In addition to the observed assumption of λ_{max} change as a stepwise function of electric field conditions in Figure 4, it was strongly confirmed that the photonic wavelength shift of mechano-chromic organogel change was in continuous manner and symmetrically reversible as shown in Figure S2(b) and(c). From the tracking plots of photonic reflection maximum λ_{max} it was found that stretching switching time was about 1.67 seconds upon applying electric field of 80 V μm^{-1}. Also, the recovering relaxation switching time was also about 1.67 seconds. Furthermore, the recovery process behavior at E-Off state was symmetry almost identical to the tuning process at E-On state, Figure S2(c). Therefore, it was believed that the photonic color tuning of organogel should be fine controllable in continuous from the assumed gentle stretching of the photonic lattice without absence of sudden wavelength jump range. From the E-on and E-off switching organogel and tracking plotting of photonic reflection band wavelength in term of λ_{max} during further time duration of 60 seconds, fully stable switching repeatability of mechano-chromic tuning in electrical methods, which is desirable for device applications, was confirmed as Figure S2(d).

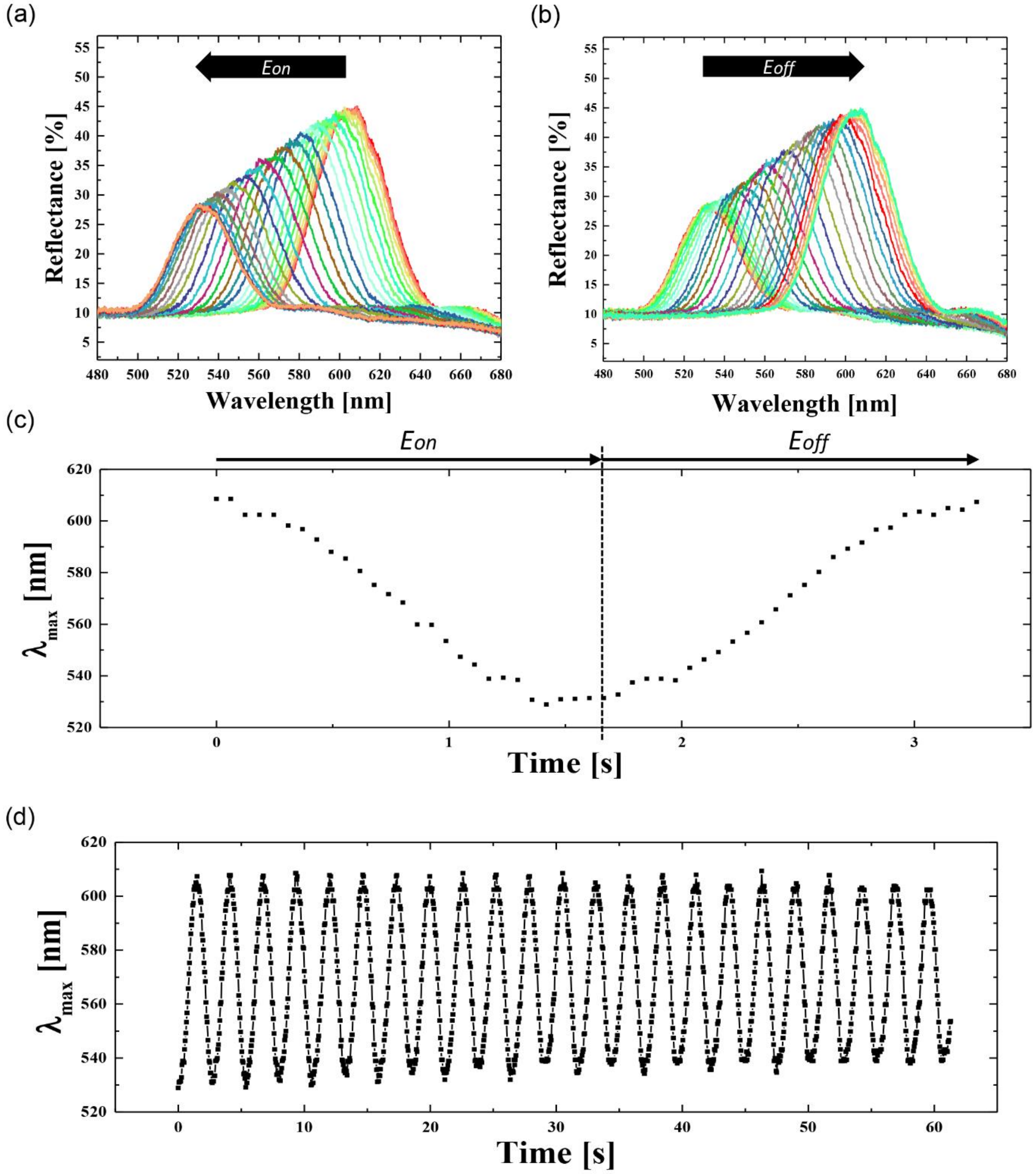

Figure S2. Reliability of electrically controlled mechano-chromic switching (a) Monitoring plot of photonic wavelength shifts of λ_{max} at E-On state during 10 seconds with electric field of 80 V·μm^{-1}. (b) Monitoring plot of recovering photonic wavelength shifts of λ_{max} at E-Off state. (c) Symmetrical electro-active mechano-chromic switching in terms of photonic reflection maximum of λ_{max} wavelength change during 60 seconds with E-On and E-Off sequenced electric field of 80 V· μm^{-1}. (d) Repeatability of electrtically stretchable mechano-

chromic switching from monitoring of photonic reflection maximum λ_{max} under repeating electric field with amplitude of 80 V· μm^{-1} and frequency of 0.3 Hz in sine wave.

Color change of uniaxial stretching mechanochromic photonic gel

To better understand the color change behaviors of mechano-chromic photonic gels, stretchable mechano chromic organogels were carefully observed between mechanical stretching method and electrically stretching method was using the in-house microscope measurement setup with motorized uniaxial stretching system and electrical field addressing system. The measurement setup was built to enable simultaneous observation of the optical status of the organogel and the spectral wavelength change while amplifying the electrical signal. The modified measurement setup, which included a microscope (BX51, Olympus), spectrometer (Flame-T, Ocean Optics), electric source composed of a function generator (AFG1022, Tektronix), oscilloscope (TBS2000B, Tektronix), and high-voltage amplifier (609B-3, Trek), are detailed in Figure S1.

In prior to observing the electrical response of the prepared electroactive photonic organogel, mechano chromic properties were investigated via direct mechanical stretching of the prepared sample (**Figure S3**). As the strain increased, lateral stretching expansion occurred, and the reflection color of red (λ_{max} = 634.41 nm) changed to green (λ_{max} = 563.98 nm). The stretched green reflection returned to the original red color as the stretching was decreased with relaxation of strain in the photonic organogels. Further observations of reflection spectrum changes of the photonic organogel while either stretching (Figure S3(a)) or relaxing (Figure S3(b)) confirmed that the photonic band changing shift was subtle and elastically recoverable. In addition, the photonic wavelength change was continuous, without any sudden wavelength change, as presented in Figure S3(c). A color change from red to orange and green was also observed as a function of mechanical strain (Figure S3(d)). A slight change in wavelength between stretching and relaxation in Figure S3(c) was assumed due to the elastic hysteresis of photonic organogels under mechanical stress. Further retuning of the shift of photonic wavelength when fully relaxed was also assumed due to the elastic inertia properties of the viscoelastic organogel film (in the same manner of relaxation of a stretched spring). The initial wavelength of maximum reflectance, λ_{max}, was 634.41 nm, whereas λ_{max} was 647.34 nm after one cycle of stretching and releasing. It is believed that the photonic structure lattice distance was also laterally elongated and compressed by the mechanically triggered stretching and relaxation.

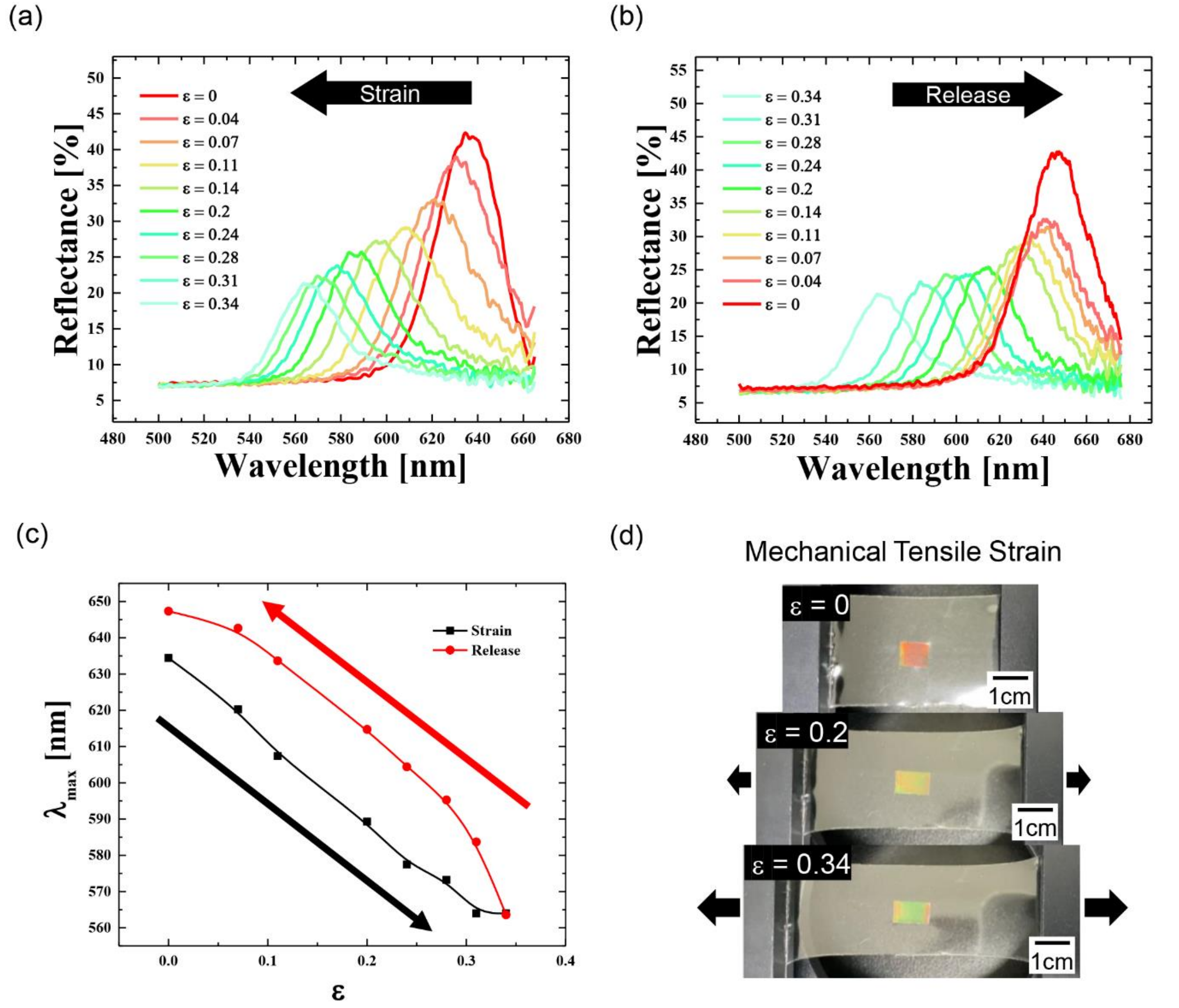

Figure S3. Mechanical stretching behavior of the photonic organogel. (a) Mechano chromic photonic wavelength shift as a function of stretching strain. (b) Mechano chromic photonic wavelength recovering shift as a function of relaxation strain. (c) Monitoring plot of the mechano chromic wavelength change in terms of photonic reflection maximum, λ_{max}, as a function of strain during the stretching and relaxation sequence. (d) Mechanical stretching induced reflection color change of the photonic organogel.

Finite Element Analysis of uniaxial and radial strain of the photonic organogel device

The FEA structure of the experimental photonic organogel device was designed to analyze the PS beads arrangement in HCP lateral and horizontal plane direction. An desinged elastomer was used for a soft substrate and a designed photonic organogel was attached to top of the elastomer. The photonic organogel consists of a backfilled organogel with HCP structured PS beads. In case of uniaxial deformation, mechanical strain was induced on the edge sides of the elastomer. Strain distribution, which was shown in color map of the FEA designed photonic organogel device, was decreased gradually from the edge of the elastomer to the center of the elastomer. By the way, the strain distribution was uniform for all the HCP lateral directions shown in Figure S4.

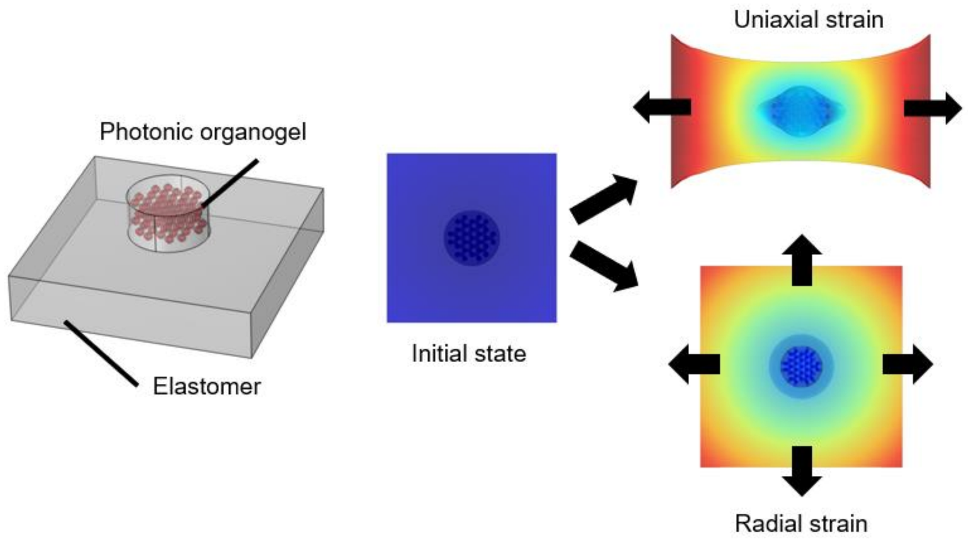

Figure S4. Strain distribution of the FEA designed photonic organogel device of uniaxial strain and radial strain.

Supporting Videos

Video S1

Macroscopic real-time video showing the structural color change of mechanically stretched photonic organogel while applying strain (ε) of 0 to 0.34 speed at the speed of 3 mm/s.

Video S2

Real-time mechanochromic photonic band spectrum switching of photonic organogel while applying strain (ε) of 0 to 0.34 speed at the speed of 3 mm/s.

Video S3

Macroscopic real-time video of the electroactive mechanochromic structural color change of the photonic organogel with 80 V μm^{-1} at a sinusoidal frequency of 0.3 Hz.

Video S4

Real-time electroactive mechanochromic photonic band spectrum switching of the photonic organogel with 80 V μm^{-1} at a sinusoidal frequency of 0.3 Hz.

Video S5

Simulation of uniaxial strain effect on the PS beads of FEA designed photonic organogel device.

Video S6

Simulation of biaxial strain effect on the PS beads of FEA designed photonic organogel device.
